# Strong Plasmon-Mie Resonance in Si@Pd Core-Ω Shell Nanocavity

**DOI:** 10.3390/ma16041453

**Published:** 2023-02-09

**Authors:** Haomin Guo, Qi Hu, Chengyun Zhang, Haiwen Liu, Runmin Wu, Shusheng Pan

**Affiliations:** 1 School of Physics and Materials Science, Guangzhou University, Guangzhou 510006, China; 2 Research Center for Advanced Information Materials (CAIM), Huangpu Research and Graduate School of Guangzhou University, Guangzhou 510555, China; 3 Key Lab of Si-Based Information Materials & Devices and Integrated Circuits Design, Department of Education of Guangdong Province, Guangzhou 510006, China

**Keywords:** femtosecond laser, Si nanosphere, nanoshell, nanoscale white light source

## Abstract

The surface plasmon resonance (SPR) and localized surface plasmon resonance (LSPR) can be used to enhance the generation of the hot electrons in plasmon metal nanocavity. In this paper, Pd nanomembrane (NMB) is sputtered on the surface of Si nanosphere (NS) on glass substrate to form the Si@Pd core-Ω shell nanocavity. A plasmon-Mie resonance is induced in the nanocavity by coupling the plasmon resonance with the Mie resonance to control the optical property of Si NS. When this nanocavity is excited by near-infrared-1 (NIR-1, 650 nm–900 nm) femtosecond (fs) laser, the luminescence intensity of Si NS is dramatically enhanced due to the synergistic interaction of plasmon and Mie resonance. The generation of resonance coupling regulates resonant mode of the nanocavity to realize multi-dimensional nonlinear optical response, which can be utilized in the fields of biological imaging and nanoscale light source.

## 1. Introduction

A nanoscale light source is an urgent need in the field of integrated photonics. The metal [1,2] and dielectric [3,4] nanospheres (NSs) are promising solutions for luminescence induced by the excited plasmon resonance and Mie resonance, respectively. The surface plasmon polaritons (SPPs) supported by noble metal NS have been a hot research topic, whose collective oscillation forms the surface plasmon resonance (SPR) [5,6]. The SPR is utilized in many fields such as encoding [7], enhancing the absorption rate of solar cells [8,9] and regulating the light polarization [10]. Furthermore, the metal@SiO_2_@dielectric core–shell nanocavity can further enhance SPR for improving the photocatalytic efficient of TiO_2_ [11], the detection efficient of RNA [12] and pH [13], and the cell imaging [14]. However, the noble metal NS can only support strong SPR, resulting in high ohmic loss, which is not conducive to integrate into micro/nanoscale optoelectronic devices. Fortunately, the dielectric NS with high refractive index is an alternative strategy that simultaneously supports both strong magnetic and electric resonance to construct nanoscale light source. Especially, the ohmic loss of dielectric NS is smaller than that of noble metal NS [15,16,17,18,19]. The high-refractive index dielectric NS [20,21,22] supports the Mie resonance, including the magnetic dipole resonance (MDR), the electric dipole resonance (EDR), the magnetic quadrupole resonance (MQR), the electric quadrupole resonance (EQR), etc. The multiphoton absorption (MPA) process appears inside the dielectric NS when the MDR is excited by the characteristic excitation wavelength of fs laser [23,24], generating a large carrier density to enhance the Auger effect which can dramatically increase the relaxation time of the carriers and result in the efficient emission of white light [25].

It is also noticed that a large number of hot electrons can be generated in the noble metal nanocavity with the assistance of SPR. Therefore, the synergistic interaction between the plasmon resonance of the noble metal nanocavity with the Mie resonance of dielectric NS is a promising new way to realize efficient nanoscale white light source. The injection of hot electrons into the conductive band (CB) of dielectric NS accelerates the particle number reversal process, increasing the probability of interband radiative transition. Hence, the dielectric@metal core–shell nanocavity can simultaneously support both the strong SPR and the Mie resonance to realize an efficient silicon-based junction nanoscale light source. For example, the resonant mode of metal nanoshell is coupled with the Mie resonance of the Si [26] and CdS [27] nanowires to concept the tunable nanoscale light source. The metal substrate affects the coupling of the hybrid system [28]. Further, the dielectric NS on metal film is constructed, and LSPR [29] is excited at the nanogap between dielectric NS and metal substrate. At the same time, the mirror effect [30,31,32,33] of metal substrate induces a mirror-EDR, which is opposite to the EDR of the Si NS. The coherent interaction of the two anti-parallel EDRs leads to the formation of a mirror-image-induced magnetic resonance at the dielectric–metal interface to further enhance the nonlinear optical response of the Si NS.

In order to demonstrate the effect of SPR and LSPR on the nonlinear optical response of Si NS, three systems are established, including Si@Pd core-Ω shell nanocavity on glass substrate (system-1), Si@Pd core-Ω shell nanocavity on Au substrate (system-2) and Si@Pd core-Ω shell nanocavity on Si substrate (system-3). By comparing those systems, an efficient double metal–semiconductor junction (MSJ) nanoscale white light source is constructed by coupling the plasmon resonance of the Pd NMB and Au substrate with the Mie resonance of Si NS (system-2). With the assistance of both SPR and LSPR, the luminescence of the Si NS dominated by the interband radiative transition transfers into the intraband transition of the hot electrons. The luminescence intensity of system-2 is 41.7% higher than that of the Si NS.

## 2. Materials and Methods

*Sample Preparation:* Si NS with different diameters were fabricated by the fs laser-induced-backward-transfer technology [34,35]. A Si wafer (thickness: 525 µm, Orientation: 111, type: N, Dopant: Sb, Ningbo Sibranch Microelectronics Technology Co., Ltd., Ningbo, China) covered with a glass (MATSUNAMI, thickness: 130 µm) was placed on the 3D electronic translation platform controlled by a computer. The fs laser delivered by a fs amplifier (Legend Elite, Coherent, San Francisco, CA, USA, central wavelength: 800 nm, pulse duration: 100 fs, repetition rate: 1 kHz, power: 4 mW) was focused on the surface of the Si substrate [36,37] in water. The solution of Si NSs prepared by fs laser ablation was dispersed on the glass, Au and Si substrates, respectively. Additionally, the Pd nanofilm is sputtered on the surface of the Si NS to form the system-1/-2/-3 by the vacuum thermal evaporation technique (ZHD-300, BEIJING TECHNOL SCIENCE CO., LTD, BEIJING, CHINA).

*Morphology characterization and Spectroscopy measurement:* The scattering cross-section and luminescence spectra of the system-1/-2/-3 were measured by using a dark-field microscope (BX53M, Olympus, Tokyo, Japan) equipped with a spectrometer (SR-500i-B1-R, Andor) and a color charge coupled device (CCD) (01-QIClick-R-F-CLR-12, QIMAGING, Surrey, BC, Canada). The 100× objective lens with a numerical aperture of 0.8 was utilized to collect the scattering and the luminescence signal excited by a fs oscillator (Mira-HP, Coherent, San Francisco, CA, USA). The integration time for the scattering spectrum measurement was chosen to be 0.6 s. The integration time for the luminescence spectrum measurement was 2 s and the gain factor was set to 20. The surface topography and the element distribution of the sample were analyzed by the scanning electron microscope (SEM, JSM-7001F, JEOL, Tokyo, Japan) and energy dispersive spectroscopy (EDS, JSM-7001F, JEOL, Tokyo, Japan).

*Numerical Simulation:* In this work, the numerical simulations are either analytically calculated based on Mie theory or numerical simulation. The Pd NMB is a Ω-type structure. The refractive index of the Pd (n_Pd_) [38] and Si (n_Si_) [39] referred to References 38 and 39. The surrounding refractive index (n_m_) was chosen to be 1. In the calculation of the scattering spectrum, the diameter of the Si NS was 200 nm while the thickness of the Pd NMB was 9 nm according to the measuring result. A mesh size as small as 1 nm was used to ensure the convergence of the numerical simulations and the achievement of accurate results. The numerical scattering cross-section $\sigma_{sca}$ was calculated according to the following formulas [19]: $\sigma_{sca}=\frac{2\pi }{k^{2}}\overset{\infty }{\underset{n=1}{\sum }}\left(2n+1\right)\left({\left|a_{n}\right|}^{2}+{\left|b_{n}\right|}^{2}\right)$, where the $k^{}=\left(2\pi n_{m}\right)/\lambda$. Based on the Mie theory, $a_{n}$ and $b_{n}$ was the EDR and MDR coefficients of the sphere in the free space, respectively.

## 3. Results

### 3.1. The Nonlinear Optical Response of Si@Pd Core-Ω Shell Nanocavity

**Figure 1:**
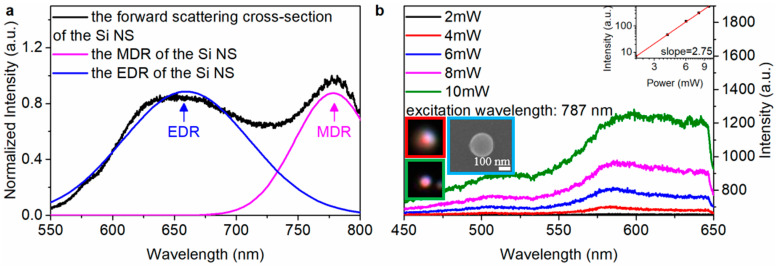
The experimental (black line) forward scattering cross-section (**a**) and the dependence of the nonlinear response on the excitation power (**b**) of Si NS. The inset shows the slope of the luminescence intensity of the Si NS versus excitation power. The red, green and cyan insets are the luminescence image, the dark field image and SEM image of Si NS, respectively.

**Figure 2:**
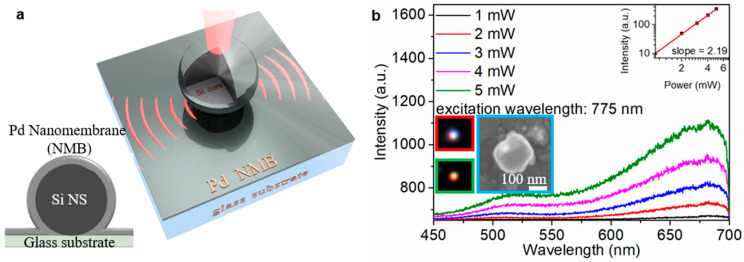
(**a**) Schematic diagram of the system-1; The insets show schematically the cross-section of the Ω-shape shell nanocavity. (**b**) The dependence of the nonlinear response on fs laser power at the characteristic excitation wavelength (MDR) of system-1; The red, green and cyan insets show the luminescence image, the dark field image and the SEM image of system-1.

The experimental forward scattering cross-section spectrum of the Si NS (Figure 1a, black line) with dimeter (d) of ~200 nm is decomposed into two obvious resonant peaks by peak analysis, including the EDR peak (Figure 1a, blue line) and MDR peak (Figure 1a, pink line). The SEM image of Si NS is shown in the cyan inset in Figure 1b. The dark field image of Si NS captured by CCD is shown in the green inset in Figure 1b. The Si NS emits fluorescence (Figure 1b, red inset) when it is excited by fs laser. The dependence of luminescence intensity of Si NS (Figure 1b) on the excitation power of fs laser is measured at the characteristic excitation wavelength of 787 nm. Figure 2a shows the schematic diagram and the cross-section diagram of the system-1, and the corresponding SEM image (d ~ 200 nm) and dark field image are shown in the cyan and green insets in Figure 2b, respectively.

**Figure 3:**
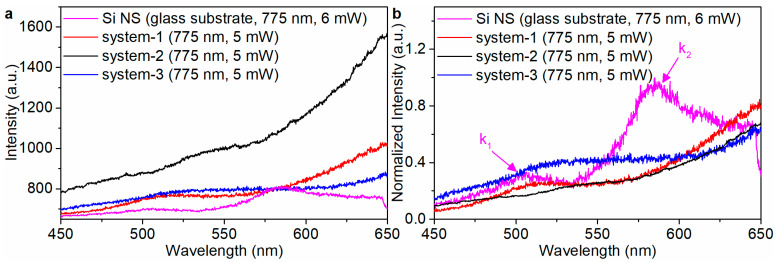
(**a**) The luminescence spectrum contrast diagram of the Si NS (pink line) and the Si@Pd core-Ω shell nanocavity at glass (red line, system-1), Au (black line, system-2) and Si (blue line, system-3) substrates; (**b**) The normalized luminescence spectrum contrast diagram of the Si NS (pink line) and the Si@Pd core-Ω shell nanocavity at glass (red line, system-1), Au (black line, system-2) and Si (blue line, system-3) substrates.

The system-1 emits fluorescence (Figure 2b, red inset) when it is excited by fs laser. The energy-dispersive spectroscopy (EDS) elemental mapping images show that the system-1 contains Si (Figure S1a), Palladium (Pd, Figure S1b) and Oxygen (O, Figure S1c) elements. The dependence of luminescence intensity of system-1 (Figure 2b) on the excitation power of fs laser is measured at the characteristic excitation wavelength of 775 nm. A linear relationship [40] log(I)=qlog(P)+const to a double-logarithmic representation of the measured luminescence intensity (I) and the excitation power (P), where the slope q represents the nonlinear power-law exponent. The slope indicates that the luminescence is dominated by intraband luminescence (IL) [1] of the hot electrons or multiphoton up-conversion luminescence (MPL) [41]. For the Si NS, the slope q is 2.75 (Figure 1b, the inset), indicating that the up-converted luminescence is dominated by 3-photon up-conversion luminescence (3PL). However, the slope q of the system-1 is 2.19 (Figure 2b, the inset), indicating that the 2-photon up-conversion luminescence (2PL) is involved in the up-converted luminescence. At the same excitation power, the luminescence intensity of the system-1 (Figure 3a, red line) is stronger than that of Si NS (Figure 3a, pink line), and the luminescence intensity of the system-1 is 8.7% (Table S1) higher than that of the Si NS. In the previous report [42], the state density of a Si nanowire (NW) is high at k_1_ (2.51) eV and k_2_ (2.18 eV).

In this paper, the 3PA process occurs in the Si NS when it is excited by NIR-1 fs laser, generating lager carriers to result in the state density high at the k_1_ (2.43 eV) and k_2_ (2.12 eV) (Figure 3b, pink line) with the assistance of the EQR and MQR. However, the state density at the k_2_ is decreased (Figure 3b, red line) when Pd NMB is coated on the surface of Si NS to form the system-1. In contrast, the MDR peak of the system-1 stretches and the EDR peak dramatically decreases when the Pd NMB is coated on the surface of the Si NS to form the system-1 (Figure S2a). The forward scattering cross section spectrum and the luminescence spectrum of another sample of the system-1 are shown in Figure S3.

### 3.2. Resonance Modes of Si@Pd Core-Ω Shell Nanocavity

**Figure 4:**
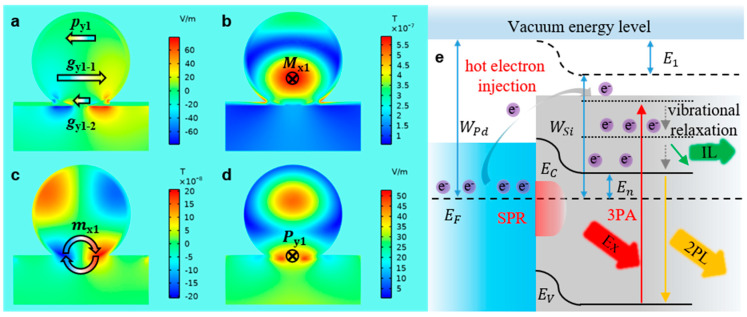
(**a**–**d**), the numerical near field distribution of the system-1 at 775 nm: the z component of the electric field distribution of the nanocavity (**a**, YZ); the total magnetic field distribution of the nanocavity (**b**, YZ); the z component of the magnetic field distribution of the nanocavity (**c**, XZ); the total electric field distribution of the nanocavity (**d**, XZ). (**e**), the energy level of the system-1.

In the numerical electric and magnetic field distribution of Si NS, under the normal incidence of a plane wave propagating in the z-direction, an electric resonance (p_y0_, Figure S4a) oriented in the y-direction. In the system-1, in the y-direction, the system-1 supports not only electric resonance (p_y1_, Figure 4a), but also two gap plasmon resonances (g_y1-1_ and g_y1-2_, Figure 4a) because the Ω-shaped SPR couples with the Mie resonance. The p_y0_ of the Si NS induces the MDR (M_x0_, Figure S4b) in the center of the Si NS. In system-1, the MDR (M_x1_, Figure 4b) moves downward inside the nanocavity with the assistance of the resonance coupling between the p_y1_, g_y1-1_ and g_y1-2_. The z component of the magnetic resonance (m_x0_) of Si NS is shown at Figure S4c. The EDR of the Si NS (P_y0_, Figure S4d) is distributed on the top and bottom of the Si NS. However, in the system-1, the g_y1-1_ enhances the magnetic resonance (m_x1_, Figure 4c) at the bottom, so the EDR (P_y1_, Figure 4d) moves downward. Therefore, the overlap degree of the MDR and EDR in the system-1 is more than that in the Si NS. Further, the work function of Pd ($W_{Pd}$, Table S2) is greater than that of the Si ($W_{Si}$, Table S2), so a Schottky MSJ is formed (Figure 4e).

### 3.3. Effect of SPR on the Nonlinear Optical Response of Si NS

The generation and injection of hot electron can be greatly promoted by the g_y1-1_ and g_y1-2_. When the system-1 is excited by NIR-1 fs laser, a 3PA process occurs in Si NS, and at the same time, the Ω-shaped SPR excites the generation of the hot electrons with energy higher than the Schottky barrier at the Pd-Si interface. The hot electrons are injected to the CB of the Si NS to accelerate electrons accumulation at the high energy level. Hence, the luminescence of the system-1 is the 2PL rather than the 3PL. The state density at the k_2_ is decreased (Figure 3b, red line) due to the intraband transition of hot electrons excited by the SPR occurs in the system-1, resulting in the attenuation of the interband radiative transition. The design of system-1 successfully demonstrates the important function of the Ω-shaped SPR to promote the generation and injection of hot electrons. However, the Ω-shaped Pd NMB generates the g_y1-1_ and g_y1-2_ in the opposite direction, causing the modes to cancel each other out and weakening the plasma resonance. Therefore, the mirror effect of the following metal nanofilm is utilized to overcome this disadvantage.

### 3.4. Influence of LSPR on Si@Pd Core-Ω Shell Nanocavity

**Figure 5:**
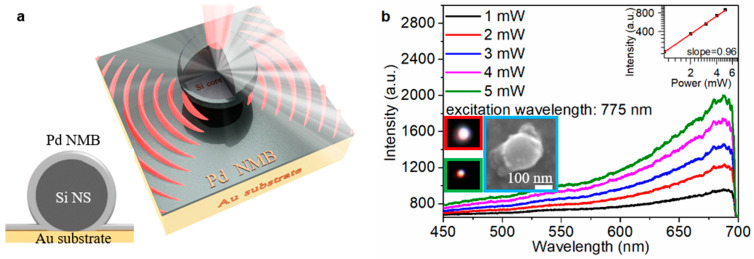
(**a**) Schematic diagram of the system-2; The insets show schematically the cross-section of the Ω-shape shell nanocavity. (**b**) The dependence of the nonlinear response on fs laser power at the characteristic excitation wavelength (MDR) of the system-2; The red, green and cyan insets show the luminescence image, the dark field image and the SEM image of system-1.

The schematic diagram of the system-2 is shown in Figure 5a. The system-2 with d ~ 200 nm (Figure 5b, cyan inset) includes Si (Figure S1d), Pd (Figure S1e) and Au (Figure S1f). The dark field image of system-2 is shown in the green inset in Figure 5b. The system-2 emits strong fluorescence (Figure 5b, red inset) when it is excited by fs laser.

The dependence of nonlinear response of the system-2 on the excitation pulse power at the MDR of the system-2 is shown in Figure 5b. At the same excitation power, the luminescence intensity of the system-2 (Figure 3a, black line) is stronger than that of the system-1 (Figure 3a, red line). The dependence of the luminescence intensity of the system-2 on the excitation power of fs laser is analyzed, providing the most direct evidence (the slope q of the system-2 is 0.96, close to 1, Figure 5b, the inset) that the luminescence of the system-2 is caused by intraband transition of the hot electrons within the CB of the Si NS. The experimental backward scattering cross-section spectra of the system-1 and system-2 are shown in Figure S2b. By comparing the backward scattering cross-section of system-1 and system-2, a new peak appears at the wavelength of 700 nm in the system-2. The electric field distribution of system-1 (Figure S2b, blue frame) and system-2 (Figure S2b, black frame) at the wavelength of 700 nm are different. Thus, the new peak at the wavelength of 700 nm is induced by the LSPR supported by Au substrate. 

**Figure 6:**
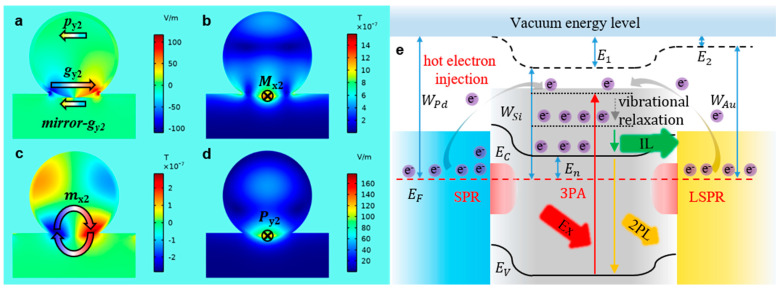
(**a**–**d**), the numerical electromagnetic field distribution of the system-2 at 775 nm: the z component of the electric field distribution of the nanocavity (**a**, YZ); the total magnetic field distribution of the nanocavity (**b**, YZ); the z component of the magnetic field distribution of the nanocavity (**c**, XZ); the total electric field distribution of the nanocavity (**d**, XZ). (**e**), the energy level of the system-2.

According to the numerical analysis, only one gap plasmon resonance occurs inside the system-2 (g_y2_, Figure 6a) so that the attenuation of plasma resonance same as system-1 is avoided. According to the dipole-image [43] mode, the mirror-g_y2_ (Figure 6a) is induced by the mirror effect of the Au substrate. Therefore, the coherent interaction of the two anti-parallel modes lead to the formation of an X-oriented MDR (M_x2_, Figure 6b), which is concentrated at the bottom of the system-2. Meanwhile, the g_y2_ enhances the magnetic resonance (m_x2_, Figure 6c) of the system-2, resulting in the Y-oriented EDR (P_y2_, Figure 6d) also to be concentrated at the bottom of the system-2. Thus, it is proposed that the excellent gain effect for the nonlinear optical response of the system-2 is due to the dual role of the g_y2_ and the mirror-g_y2_, which makes the EDR and MDR of the Si NS coincide. The EDR and MDR of the Si NS can be excited simultaneously when the system-2 is excited by NIR-1 fs laser, resulting in the gain effect for the nonlinear optical response of the system-2 greater than that of the system-1.

Meanwhile, only one Schottky barrier cannot form a potential well to limit the escape of the hot electrons. In the system-2, the work function ($W_{Au}$) of Au is larger than that of the Si, another MSJ can be formed at the Si-Au interface. The system-2 with two Schottky barriers forms a potential well that limits the escape of the hot electrons (Figure 6e), which dramatically increases the accumulation of electrons at the high energy level. When the system-2 is excited by NIR-1 fs laser, the 3PA process occurs in the Si NS, and at the same time, the Ω-shaped SPR and LSPR excite the generation of the hot electrons on the Ω-shaped Pd NMB and the Au substrate, respectively. Under the combined action of Ω-shaped SPR and the LSPR, the intraband transition of the hot electrons is stronger than the interband radiative transition of the hot electrons, resulting the luminescence of system-2 is dominated by the IL of the hot electrons, and the state density at k_1_ and k_2_ is simultaneously decreased (Figure 3b, black line). The scattering cross-section and luminescence spectra of another sample of the system-2 are shown in Figure S5. To further demonstrate that the intraband transition competes with the interband radiative transition, the Au substrate is replaced with Si substrate to form the system-3. A detailed analysis is provided in Section B of the Supplementary Information (Figures S6–S9).

## 4. Conclusions

In summary, the Ω-shaped Pd NMB and Au substrate form Schottky MSJ with the Si NS, respectively. When the system-2 is excited by the NIR-1 fs laser, the 3PA process occurs in the Si NS. At the same time, the excited Ω-shaped SPR and LSPR simultaneously generate the hot electrons on the Pd-Si and Si-Au interfaces, respectively. Under the combined action of Ω-shaped SPR and the LSPR, the injection of hot electron is stronger than the up-conversion process, dramatically increasing the accumulation of electrons at the high energy level. Hence, the intraband transition is stronger than the interband radiative transition, resulting in the luminescence of the system-2 dominated by IL. The decrease in density of state at k_1_ and k_2_ proves that the injection of the hot electrons results in a significant change in carrier density distribution. Meanwhile, The MDR and EDR of the Si NS overlap well in system-2 with the assistance of the Ω-shaped SPR and LSPR. The MDR and EDR are simultaneously excited by the NIR-1 fs laser to achieve multi-dimensional nonlinear optical response of the Si NS. At the same excitation power, the luminescence intensity of system-2 is stronger than that of Si NS (41.7%), system-1 (30.4%) and system-3 (31.8%). It is proved that a laser driven double MSJ nanostructure (system-2) can efficiently emit light by matching the plasmon resonance of the Pd NMB and Au substrate with the Mie resonance of Si NS. The optical manipulation of plasmon-Mie resonance coupling provides a new strategy for controlling light-matter interaction and presents the application prospect of dielectric–metal hybrid nanocavity as integrated efficient nanoscale white light source.

## Data Availability

Data underlying the results presented in this paper are not publicly available at this time but may be obtained from the authors upon reasonable request.

## Supplementary Materials

The following supporting information can be downloaded at: https://www.mdpi.com/article/10.3390/ma16041453/s1, Figure S1 contains the element distribution of system-1 and system-2. Figure S2 contains the scattering cross-section spectra of the system-1 and system-2. Figure S3 contains the cross-section spectra and the dependence of the nonlinear response on the excitation energy of system-1 (second sample). Figure S4 contains the numerical simulation of electric and magnetic field at 775 nm (Si NS on glass substrate). Figure S5 contains the cross-section spectra and the dependence of the nonlinear response on the excitation energy of system-2 (second sample). Figure S6 contains the SEM image and element distribution of system-3. Figure S7 contains the cross-section spectra and the dependence of the nonlinear response on the excitation energy of system-3 (first sample). Figure S8 contains the electric, magnetic field and energy level of system-3. Figure S9 contains the cross-section spectra and the dependence of the nonlinear response on the excitation energy of system-3 (second sample). Table S1 contains the integration area of luminescence of four systems. Table S2 contains the work function of the Pd, Si and Au.
