# Supplementary material for: Strong Plasmon-Mie Resonance in Si@Pd Core-Ω Shell Nanocavity
Source: Materials (Basel). 2023 Feb 9;16(4):1453. doi: 10.3390/ma16041453 (PMC9961871; doi:10.3390/ma16041453)
Supplement: Supplementary file 1 [file materials-16-01453-s001.zip › materials-2188991-supplementary.pdf]

Supporting Information

Strong Plasmon-Mie Resonance in Si@Pd Core-Ω Shell Nanocavity

Haomin Guo [1], Qi Hu [1,2], Chengyun Zhang [1,2,3,*], Haiwen Liu [1], Runmin Wu [1] and Shusheng Pan [1,2,3,*]

[1] School of Physics and Materials Science, Guangzhou University, Guangzhou 510006, China
[2] Research Center for Advanced Information Materials (CAIM), Huangpu Research and Graduate School of Guangzhou University, Guangzhou 510555, China
[3] Key Lab of Si-Based Information Materials & Devices and Integrated Circuits Design, Department of Education of Guangdong Province, Guangzhou 510006, China
* Correspondence: chyzhang@gzhu.edu.cn (C.Z.); sspan@gzhu.edu.cn (S.P.)

Section A: the Supplementary Information of the Si NS, system-1 and system-2

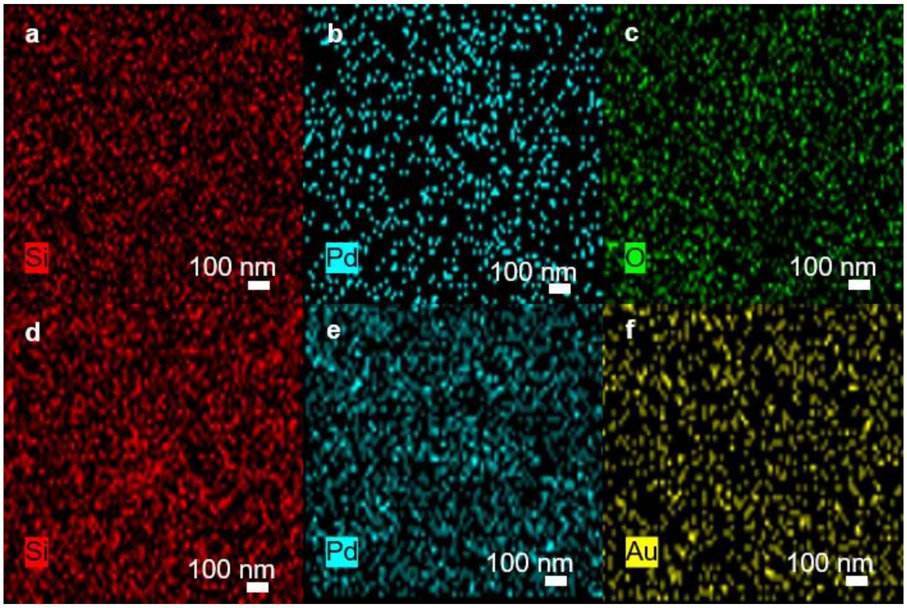

Figure S1. The element distribution of Si (**a**), Pd (**b**), O (**c**) of the system-1; The element distribution of Si (**d**), Pd (**e**) and Au (**f**) of the system-2.

Table S1. The integration area of luminescence intensity of the four samples.

Sample	Integration area (wavelength range: 460 nm - 640 nm)
Si NS	131,259
Si@Pd core-Ω nanoshell nanocavity (glass substrate)	142,729
Si@Pd core-Ω nanoshell nanocavity (Au substrate)	186,056
Si@Pd core-Ω nanoshell nanocavity (Si substrate)	141,177

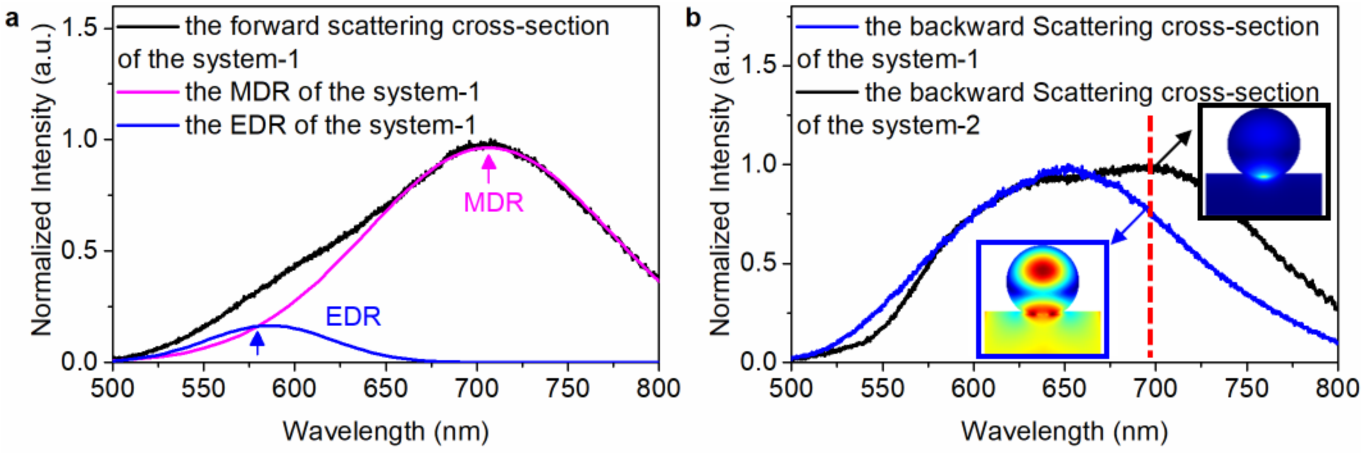

Figure S2. (b) The forward scattering cross-section spectra of the system-1(black line), the MDR (pink line) and EDR (blue line) peak of system-1; (**b**) The backward scattering cross-section spectra of the system-1 (blue line) and system-2 (black line); The electric field distribution of the system-1 (blue frame) and system-2 (black frame).

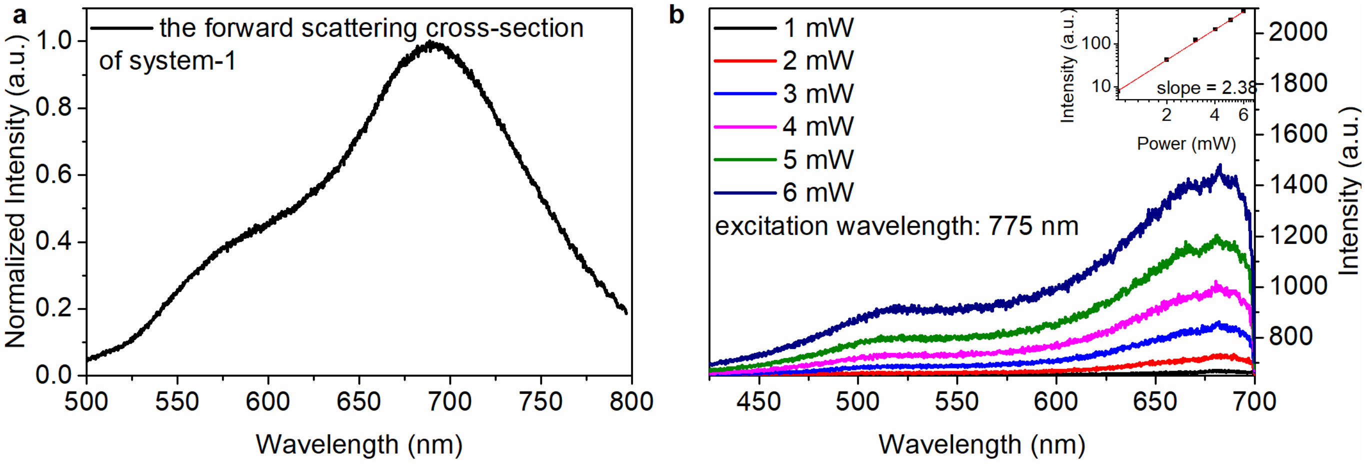

Figure S3. The forward scattering cross-section (**a**) and the dependence of the nonlinear response on the excitation energy (**b**) of the system-1; The inset: the slope of the luminescence intensity of the system-1 versus excitation power. (the second sample of the system-1).

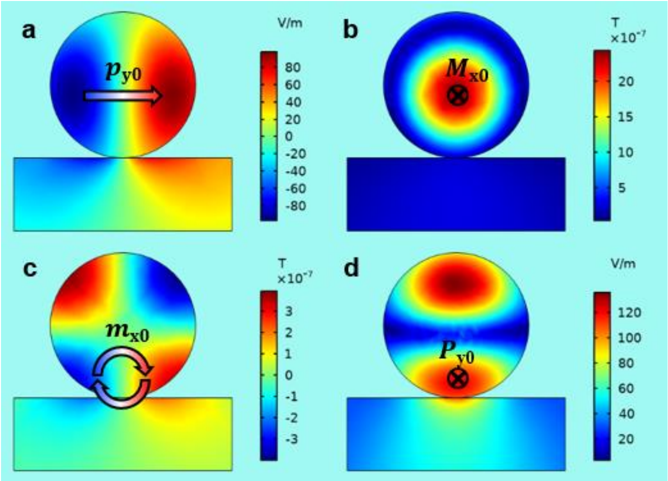

Figure S4. The numerical simulation of the electric and magnetic field distribution at the 775 nm. (**a**–**d**)

The numerical simulation of the Si NS on the glass substrate: the z component of the electric field distribution of the nanostructure (**a**, YZ); the total magnetic field distribution of the nanostructure (**b**, YZ); the z component of the magnetic field distribution of the nanostructure (**c**, XZ); the total electric field distribution of the nanostructure (**d**, XZ).

The work function of Pd (W_{Pd}) is 5.12 eV, and the affinity potential energy of Si is 4.05 eV. The resistivity of silicon used in this work is 0.01-0.018 Ω·cm, and the distance between Fermi level distance and conduction band bottom of silicon is calculated as 0.108 eV-0.125 eV by the formula: $E_n = k_0 T \ln(N_c/n_0)$. The work function of Si (W_{Si}) is 4.158 eV-4.175 eV. And the work function of Au (W_{Au}) is 5.1 eV.

Table S2. The work functions of the Pd, Si and Au.

W_{Pd}	5.12 eV
W_{Si}	4.158 eV–4.175 eV
W_{Au}	5.1 eV

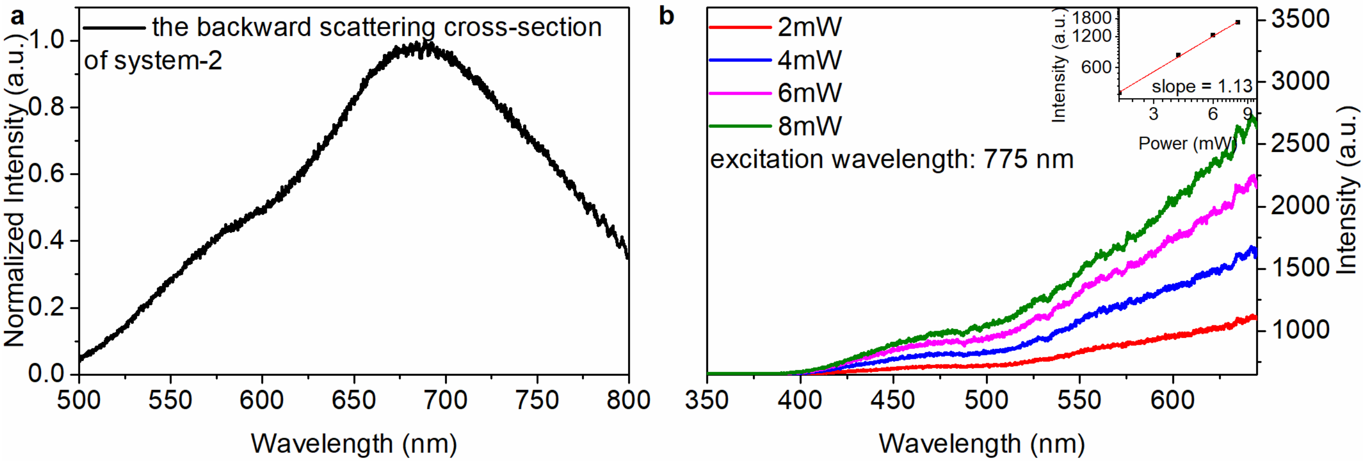

Figure S5. The backward scattering cross-section (a) and the dependence of the nonlinear response on the excitation energy (**b**) of the system-2; The inset: the slope of the luminescence intensity of the system-2 versus excitation power. (the second sample of the system-2).

Section B: Effect of Si substrate on the nonlinear response of Si@Pd core-Ω shell nanostructure

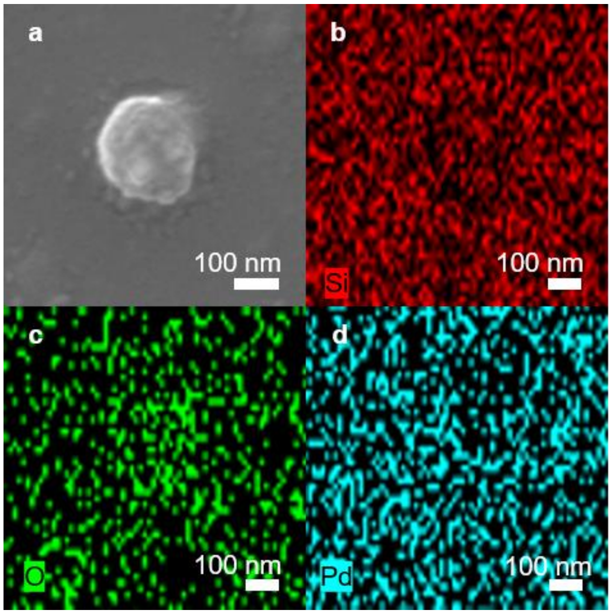

Figure S6. The SEM and EDS images. (**a**) the SEM images of the system-3; (**b**–**d**) the element distribution of Si (**b**), O (**c**), Pd (**d**) of the system-3.

The surface morphology of the system-3 with d ~ 200 nm is shown in Figure S6a. At the same time, the EDS of the system-3 is measured to analyze the elements, including the Si (Figure S6b), the O (Figure S6c) and the Pd (Figure S6d) elements. When the Au substrate is replaced by Si substrate, the mirror effect caused by Au substrate disppears. The experimental backward scattering cross-section spectrum of the system-3 is shown in Figure S7a. According to the analysis of the luminescence of the system-3, the sploe of the system-3 is 1.64 (Figure S7b, inset), indicating that the luminescence of the system-3 is 2PL. At the same excitation power, the luminescence intensity of the system-3 (Figure 3a, bule line) is the weakest among the three systems. And then, the numerical near-field diagram of the system-3 is plotted to analyze the influence of the Si substrate.

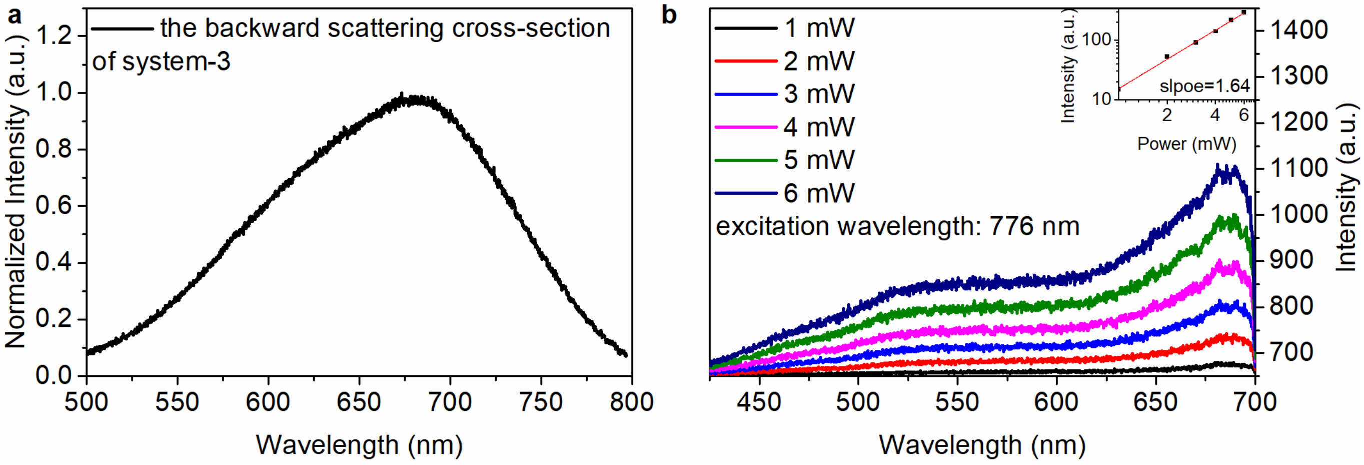

Figure S7. The backward scattering cross-section (**a**) and the dependence of the nonlinear response on the excitation energy (**b**) of the system-3; The inset: the slope of the luminescence intensity of the system-3 versus excitation power.

The p_{y3} mode is induced by the Mie resonance and the g_{y3} mode is induced by the Ω-shaped SPR (Figure S8a). However, a new mode (s_{y3}, Figure S8a) appeares in the Si substrate because of the Si substrate with high refractive index. The s_{y3} mode orientation is same as the g_{y3} mode. So the MDR simultaneously exists in the the nanocavity and the Si substrate (M_{x3}, Figure S8b) under the coupling of the p_{y3} mode, g_{y3} mode and s_{y3} mode. And the m_{x3} mode (Figure S8c) is also enhanced by the g_{y3} mode and s_{y3} mode. Therefore, the EDR is distributed at the center and bottom of the nanocavity (P_{y3}, Figure S8d). The MDR of the system-3 is distributed in the Si substrate, indicating that there is energy transfer between the Si@Pd core-Ω shell nanocavity and the Si substrate. The energy level analysis of the system-3 (Figure S8e) clearly demonstrates this phenomenon. When the system-3 is formed, the MSJ appears at the Pd-Si interface and the energy band of the Si NS overlaps with that of the Si substrate. The Ω-shaped SPR excites the generation of the hot electrons on the Ω-shaped Pd nanoshell. The hot electrons are injected to the CB of the Si NS to accelerate electrons accumulation, and the intraband transition formed in the CB of the Si NS leads to the attenuation of radiative transition.

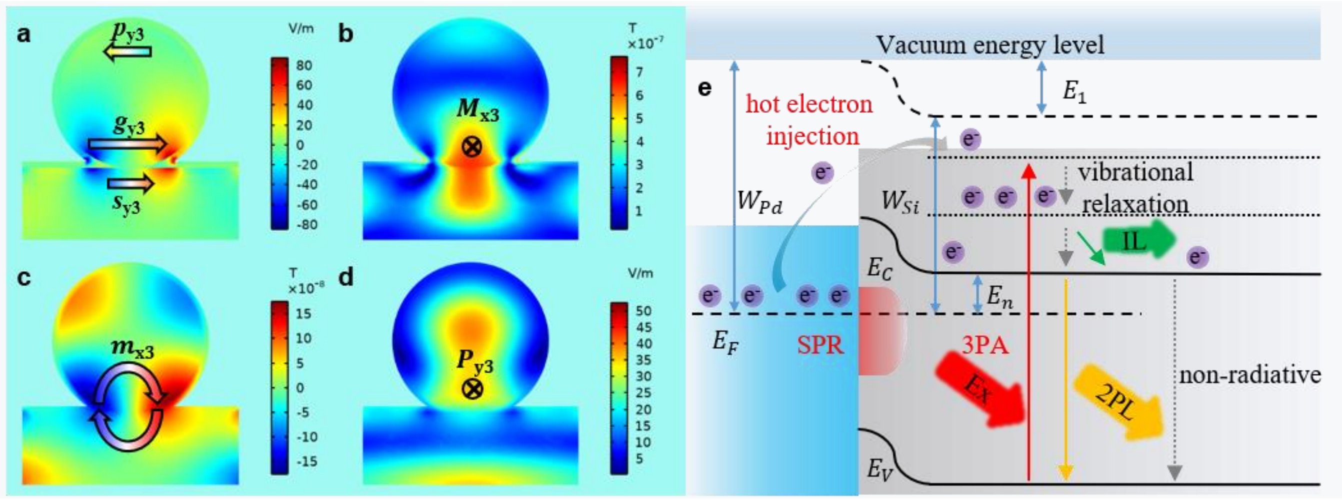

Figure S8. a–b, the numerical electric and magnetic field distribution of the system-3: the z component of the electric field distribution of the nanocavity (**a**, YZ); the total magnetic field distribution of the nanocavity (**b**, YZ); the z component of the magnetic field distribution of the nanocavity (**c**, XZ); the total electric field distribution of the nanocavity (**d**, XZ). (**e**) the energy level of the system-3.

Due to the overlap of energy band between the Si NS and the Si substrate, the hot electrons will flow into Si substrate. Moreover, the Si substrate is a bulk material which cannot be excited by NIR-1 fs laser and thus consumes electrons through thermal non-radiative transition rather than radiative transition. The slope of the system-3 is 1.64, indicating the existence of the up-conversion process, the hot electrons injection and the energy band overlap. Therefore, the luminescence intensity of the system-3 is weaker than that of the system-1 (1.1 %) and system-2 (31.8 %). The luminescence spectra and the scattering cross-section spectrum of another sample of the system-3 are shown in Figures S9.

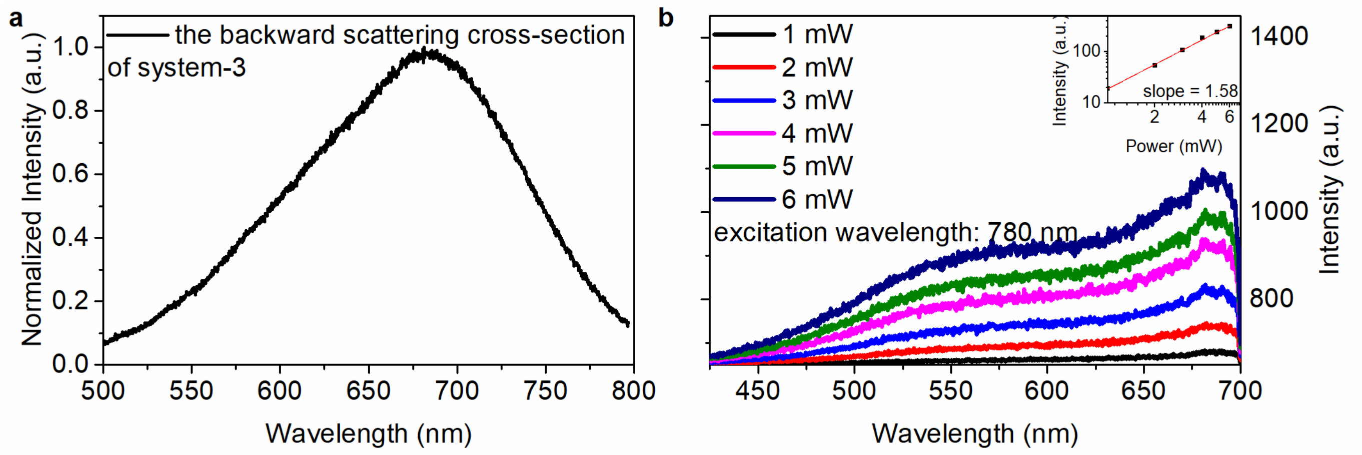

Figure S9. The backward scattering cross-section (**a**) and the dependence of the nonlinear response on the excitation energy (**b**) of the system-3; The inset: the slope of the luminescence intensity of the system-3 versus excitation power. (the second sample of the system-3).
